# Role of suture diameter and vessel insertion position in the establishment of the middle cerebral artery occlusion rat model

**DOI:** 10.3892/etm.2013.1046

**Published:** 2013-04-03

**Authors:** QIQIANG TANG, RUODONG HAN, HAN XIAO, LILI SHI, JILONG SHEN, QINGLI LUN, JUN LI

**Affiliations:** 1 Department of Neurology, Affiliated Provincial Hospital of Anhui Medical University; Hefei, Anhui 230032, P.R. China; 2 Institute of Clinical Pharmacology, Anhui Medical University, Key Laboratories of Zoonoses and Pathogen Biology; Hefei, Anhui 230032, P.R. China; 3 School of Pharmacy, Anhui Medical University, Hefei, Anhui 230032, P.R. China

**Keywords:** suture method, middle cerebral artery occlusion model, rat model

## Abstract

The aim of the present study was to explore the role of suture diameter and vessel insertion position in the preparation of the middle cerebral artery occlusion (MCAO) rat model. A total of 84 Sprague-Dawley rats (weighing 250–300 g) were randomly divided to three groups: group A (type 1.0, suture diameter 0.16–0.17 mm and tip 0.21–0.22 mm); group B (type 2.0; suture diameter, 0.22–0.23 mm; tip, 0.27–0.28 mm); and group C (type 3.0; suture diameter, 0.28–0.29 mm; and tip, 0.33–0.34 mm). The animals in each group were then subdivided into two subgroups, one of which received a nylon line inserted through the external carotid artery (ECA insertion), while the other received the nylon line through the common carotid artery (CCA insertion) subsequent to a middle or lateral neck incision. The neurological deficit score was evaluated at 4, 8, 24, 48 and 72 h post-surgery. The ischemic brain tissue was stained by 2,3,5-triphenyltetrazolium chloride (TTC) to evaluate the extent of the infarct volume. The cerebral edema rate, cerebral infarction volume rate, relative standard deviation (RSD) of the cerebral infarction rate and the success rate were also assessed. The rectal temperature, PaO_2_, PaCO_2_, pH, blood pressure and blood glucose levels were controlled and did not vary between the group types. The results suggested that suture diameter and insertion route affected the infarct volume and success rate in the establishment of the suture MCAO rat model. Furthermore, the MCAO model with a 0.22–0.23 mm diameter suture and CCA insertion route provided the highest success rate in the SD rats.

## Introduction

Acute ischemic cerebrovascular disease is the most common of the nervous vascular diseases. According to the Framingham epidemiological studies, 65% of stroke cases result from an occlusion of the middle cerebral artery (1). Thus, the ischemia model created through middle cerebral artery occlusion (MCAO) is often employed in studies concerning cerebral ischemia diseases. The suture method is more widely used than other methods (2,3); it avoids opening the skull, leads to less injury, indicates the precise location of the occlusion and controls the duration of ischemia and reperfusion (4). Researchers in this field are refining a suitable method by modifying the end of the line, implantation depth and occlusion time to improve the MCAO model. However, the extent of cerebral infarction remains uncertain due to the inter-individual anatomical variety of cerebrovascular structures. The success rate and reliability of the model are affected by numerous factors, including strain, gender, weight, age, incision, ligation, quality and hardness of the materials, shape of the end of the line and the anesthetics used. Currently, there are no systematic studies on the size of the nylon line and particularly on the location of the neck incision and the vessel ligation. The tip diameter of the suture varies largely from 0.18 to 0.30 mm (5–7). The present study was performed to systematically demonstrate the effects of suture diameter (type 1.0, 2.0 and 3.0, suture diameters ranging from 0.16 to 0.28 mm), the arterial location of the ligation and the surgical approaches. The focal MCAO model was used in an effort to identify a simple and reliable focal cerebral ischemia model for use in clinical and experimental studies.

## Subjects and methods

### Animals

The animals were purchased from the Vital River Laboratories (VRL; Beijing, China). The rats were kept in isolators at a temperature of 23±20°C and a relative humidity of 55±10%, on a 12 h dark/light cycle (06:00–18:00) with air exchanged ≥12 times per hour.

The animals were treated humanely, according to the Animal Ethics Procedures and Guidelines of the People’s Republic of China and the study was approved by Anhui Medical University.

### Experimental design

A total of 84 adult male Sprague-Dawley rats weighing 250–300 g were randomly divided into three groups with 28 rats each according to the type of nylon line: Group A (type 1.0; suture diameter, 0.16–0.17 mm; and tip, 0.21–0.22 mm); group B (type 2.0; suture diameter, 0.22–0.23 mm; and tip, 0.27–0.28 mm); and group C (type 3.0, suture diameter, 0.28–0.29 mm; and tip, 0.33–0.34 mm). The animals in each group were then subdivided into two subgroups: In the 1st groups (A1, B1 and C1) the nylon line was inserted through the common carotid artery (CCA); and in the 2nd group (A2, B2 and C2) the nylon line was inserted through the external carotid artery (ECA). In each of the subgroups, half of the animals were subjected to incisions in the middle of the neck and the other half were subjected to lateral incisions (subgroups 1 and 2).

### Apparatus and reagents

The nylon line was purchased from Seaguar (Tokyo, Japan), the 2,3,5-triphenyltetrazolium chloride (TTC) was purchased from Sigma (St. Louis, MO, USA) and the electronic vernier caliper was purchased from Hangzhou Meka Tools Co., Ltd (Hangzhou, China).

### Occluding suture preparation

Three types of nylon lines were prepared according to the method reported by Ma *et al*(8) with minor modifications. A 6–10 cm line was then curved by knotting it at a site between one-third and one-half of the length. A 170° angles was formed automatically between the two sides of this knot, 20 and 10 mm lateral to the knot. The site 5 mm from the end of the 20-mm segment was embedded in silica gel. The diameter of the tip was precisely controlled by an electronic vernier caliper to fit one of the three size ranges: 0.21–0.22 mm, 0.27–0.28 mm or 0.33–0.34 mm. The prepared occluding sutures were sterilized by UV for storage and treated sequentially with iodophor, alcohol and saline prior to use.

### Operation

#### Incision and insertion

The two approaches used in this study were those of anterior middle and lateral incisions with the line inserted through one of two arteries, the right ECA or the right CCA, respectively. All male SD rats were anesthetized with 10% chloral hydrate. With the use of aseptic surgical techniques, catheters were inserted into the right femoral artery and vein to measure the arterial blood pressure (MABP) and arterial blood gases (pH, PaCO_2_ and PaO_2_). The rectal temperature was maintained at 36.5–37.5°C using an electric blanket during the surgery. These physiological parameters were monitored prior to, during and subsequent to the MCAO.

#### ECA insertion

This method was based on the study by Longa *et al*(9) with slight modifications. The animals were anesthetized by an intraperitoneal injection of 10% chloral hydrate (0.4 ml/100 g) following a 24-h fast. A 20-mm middle or lateral incision was made in the neck following skin preparation, sterilization and exposing neck fully. The subcutaneous tissue was dissected bluntly to expose the right CCA and to isolate the internal carotid artery (ICA), ECA and vagus nerve along the CCA. The ECA was ligated at its first bifurcation and a small opening was made on the proximal ECA at the site that was 4–5 mm distal to the CCA bifurcation, subsequent to temporarily blocking the blood flow in the CCA and ICA with an artery clip. A silicone-coated nylon suture was introduced into the ECA through the small opening. A 6-0 silk suture was placed loosely around the ECA near the small opening to avoid blood backflow through the ECA and maintain the mobility of the intraluminal nylon suture. Caution was taken so as not to traumatize the arterial wall. The ECA was cut off between the small opening and the first nylon tie, then the artery clip was withdrawn. The ECA was pulled in the direction of the heart and the ECA and ICA were arranged in a line and the suture was continuously pushed into the ICA until it met resistance. The 6-0 silk suture was tightened around the ECA to fix the occluding suture. Typically, an 18±2 mm length of line is pushed into the vessels (10). If the length is <15 mm, the occluding suture must be pulled out and inserted again. The extravascular nylon suture was cut off with micro scissors. The incision was sutured with a 3-0 silk suture and then the rat was placed in a 35°C nursing box to recover from the anesthesia prior to being returned to the cage.

#### CCA insertion

This method was based on the study by Engel *et al*(11) with minimal modifications. The surgical preparation was the same as previously described. Following the exposure of the CCA, ICA, ECA and vagus nerve, the ECA was ligated at its first bifurcation. The CCA was then ligated 5 mm from its bifurcation to the ECA and ICA, following the separation of the ICA and vagus nerve and the temporary blockade of the blood flow in the ICA with an artery clip. A small hole was opened in the CCA 2–4 mm from the bifurcation and the occluding suture was inserted into the ICA. A 6-0 silk suture was loosely tied around the CCA to avoid any blood backflow from the small opening. The artery clip was removed and the blood flow was released, then the nylon suture was pushed forward until it met resistance. The loose silk suture was lightened to fix the intraluminal nylon suture. In this procedure, the 18±2 mm nylon suture was pushed into the vessels to avoid having to pull out the nylon suture embolism and insert it again.

### Evaluation index and method

#### Neurological deficit function

The severity of the neurological deficit was assessed at 4, 8, 24, 48 and 72 h post-surgery according to the Zea-Longa neurological deficit score. A score of 0 was given to normal animals with no signs of neurological deficit. A score of 1 was given to those with mild neurological disorders, including dysfunction in stretching the left anterior limb. A score of 2 was given to animals that intorted the left anterior limb and adducted the shoulder when they were lifted by the tails and also turned left when they walked. A score of 3 was given to animals with neurologic abnormalities so severe that they fell to the left side when they walked. Rats with a score of 4 did not walk spontaneously and had a depressed level of consciousness.

#### Detecting cerebral ischemia volume and edema

All surviving rats were sacrificed 72 h post-surgery in accordance with a previous study (12). The brains were dissociated on ice and boxed into a special brain mold following the depletion of the cerebellum and brain stem. The brains were cut into a series of 2-mm thick coronal slices and incubated in 1% TTC for 20 min at 37°C and then in 4% paraformaldehyde for 30 min. The cross-sectional area of the infarction and the non-infarcted tissue in each brain slice was measured using Image J analysis software (version 1.6 NIH). The infarct volume was indirectly calculated according to the following formula: Infarct volume = (Σ infarct area × thickness) / (Σ whole area × thickness) × 100. Extent of edema = (volume of right hemisphere - volume of left hemisphere / volume of left hemisphere) × 100. To calculate the relative standard deviation (RSD), first the whole brain was weighed (Tm), then the infarcted region was dissociated and weighed (Pm). The RSD of the infarction = (Pm / Tm) × 100. Success rate = number of successful models / total number of models × 100.

#### Statistical analysis

Continuous variables are shown as the mean ± SD. Statistical analysis was carried out using SPSS 13.0 software. A comparison of the differences between the experimental groups was performed using the one-way ANOVA test. Rate comparisons were analyzed using the Chi-square (χ^2^) test. P<0.05 was considered to indicate a statistically significant difference.

## Results

### 

#### General features and physiological parameters of the animals

Several systemic factors may affect the preparation of a MCAO rat model. These factors include rectal temperature, blood pressure and blood pH. A total of 84 adult male SD rats weighing 250–300 g were used in the present study. The physiological variables were monitored 10 min prior to the onset of MCAO (prior to ischemia), 10 min subsequent to the onset of MCAO (during ischemia) and 30 min subsequent to the end of MCAO (subsequent to ischemia) in groups A, B and C. All data were kept within normal physiological limits prior to, during and subsequent to ischemia. There were no significant differences in weight, rectal temperature, PaO_2_, PaCO_2_, pH, blood pressure or blood glucose among the three groups (P>0.05).

#### Cerebral edema rate, cerebral infarction rate and RSD

**Figure 1:**
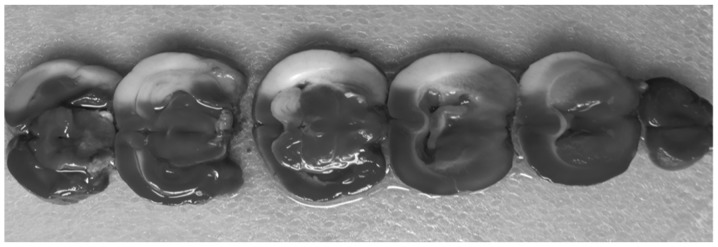
Rat brain slices (2-mm thick) stained by 1% TTC solution 72 h subsequent to the MCAO experiment. Normal brain tissue was visualized as red, while infarct brain tissue was white. TTC, 2,3,5-triphenyltetrazolium chloride; MCAO, middle cerebral artery occlusion.

Fig. 1 shows typical images of the TTC-stained sections of the infarct and normal tissue. There was a clear border between the red and white matter; the ischemic region was white and the normal tissue was rose-colored. The cerebral edema rate, cerebral infarction rate and RSD in all groups were evaluated subsequent to MCAO. There was no significant difference in the cerebral edema rate among groups A, B and C (F=1.426; P=0.243). The cerebral infarction rates in groups A, B and C were 20.723±1.419, 21.063±1.163 and 21.614±1.067%, respectively; these values were significantly different (F=7.024; P=0.001). A Student-Newman-Keuls(S-N-K) comparison showed that the cerebral infarction rate was significantly different between groups A and C or B and C (P<0.05), but not between groups A and B (P>0.05). There also were no significant differences in the cerebral infarction rate between the subgroups or small subgroups in the 3 groups (P>0.05). The RSDs of the cerebral infarction rates in groups A, B and C were 6.85, 5.52 and 4.94%, respectively.

#### Pathomorphology of the rat brain

**Figure 2:**
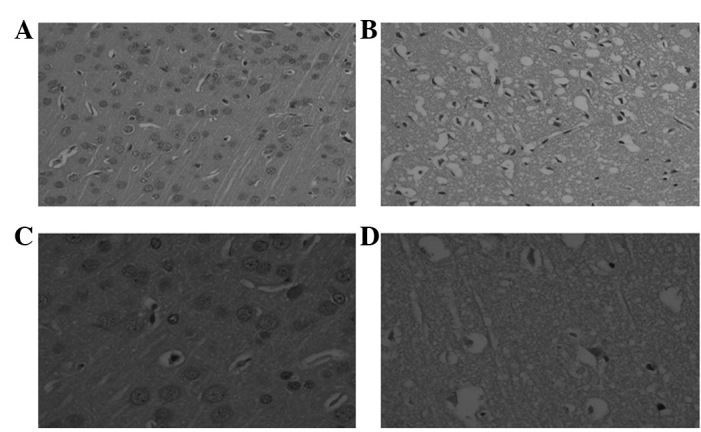
HE staining was carried out 72 h subsequent to the MCAO experiment. (A) Low-power photomicrograph (×200) of the normal zone. (B) High-power photomicrograph (×400) of the normal zone. (C) Low-power photomicrograph (×200) of the infarct border zone. (D) High-power photo-micrograph (×400) of the infarct border zone. MCAO, middle cerebral artery occlusion.

At 72 h post-surgery, the brains were subjected to TTC staining, 4% paraformaldehyde treatment, dehydration and paraffin embedding. Coronal sections were observed under the microscope following the creation of the pathological slide and HE staining. The normal zone is shown in Fig. 2A and B; the neural cells had normal laminar structures and there was no edema, abnormal vasculature or inflammatory cell infiltration. However, on the artery-blocked side shown in Fig. 2C and D, the infarcted region displayed liquefaction, necrosis and inflammatory cell infiltration. The neural cells swelled then broke down into fragments, underwent karyopyknosis and disappeared. The tissue around the necrotic region loosened, the cells swelled and the perivascular space enlarged.

#### Nervous behavior scoring

When evaluated using neurological deficit scoring, 8 rats received a score of zero: 7 (87.5%), 1 (12.5%) and 0 (0%) in groups A, B and C, respectively. Seven animals received a score of 4: 0 (0%), 1 (14.3%) and 6 (85.7%) in groups A, B and C, respectively. A score of 1 was given to 15 animals, including 9 (60.0%) in group A, 4 (26.7%) in group B and 2 (13.3%) in group C. A total of 33 rats achieved a score of 2, including 7 (21.2%) in group A, 15 (45.5%) in group B and 11 (33.3%) in group C. Only 3 rats scored 3 marks: 0 (0%) in group A, 1 (33.3%) in group B and 2 (66.7%) in group C. In total, 18 animals died during the surgery; 2 (11.1%) from excessive anesthesia, 5 (27.8%) from subarachnoid hemorrhage (SAH), 2 (11.1%) from dyspnea, 6 (33.3%) from epilepsy and 3 (16.7%) for no clear reason. These 18 animals were not included in the neurological deficit scoring.

**Figure 3:**
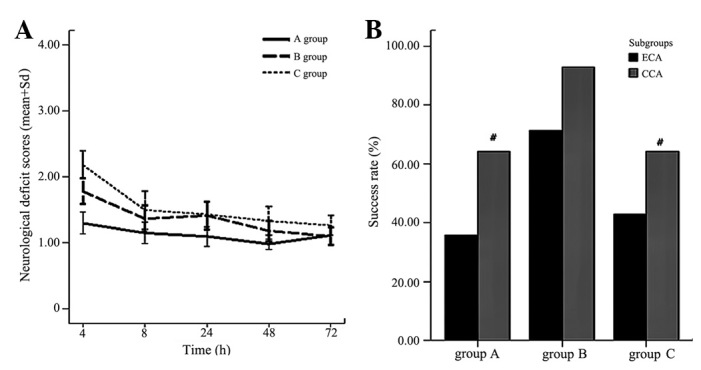
(A) Neurological scores at 4, 8, 24, 48 and 72 h subsequent to the MCAO in groups A, B and C. Data are expressed as the mean ± SD. Statistical analysis was carried out with a one-way ANOVA. (B) The success rate of the MCAO model was determined at 72 h subsequent to the ischemic insult (n=28 per group). Statistical analysis was carried out using the Chi-square (χ^2^) test. The success rate was higher in the CCA subgroup than in the ECA subgroup of each main group. However, the difference was not significant (P>0.05). ^#^P<0.05 vs. group B. MCAO, middle cerebral artery occlusion; CCA, common carotid artery; ECA, external carotid artery.

The variation curves of the Zea-Longa scores at 4, 8, 24, 48 and 72 h subsequent to MCAO in all three groups are shown in Fig. 3A. There were no significant differences among the 5 time-points in group A (F=0.393; P=0.814). However, the scores in group B differed significantly at 4, 8, 24, 48 and 72 h (F=16.315; P=0.000). A marked difference was observed between hour 4 and the other time-points subsequent to MCAO (P<0.05), but not among the 8, 24, 48 and 72 h time-points (P>0.05). Similar to group B, group C displayed scores with a significant difference abetween hour 4 and the subsequent time-points (F=13.911; P=0.000). A paired comparison showed that the scores at 4 and 8 h were significantly different from those observed at 24, 48 and 72 h (P<0.05). There were no significant differences among the 8, 24, 48 and 72 h time-points subsequent to MCAO (P>0.05).

#### Success rate

The criteria for a successful MCAO model was that the animals scored 1, 2 or 3 marks in the neurological deficit scoring at 4 h post-surgery and survived for 72 h. Also, TTC staining indicated an evident infarcted region. Fig. 3B shows that the success rate in group B was the highest while that of group C was the lowest; there was a significant difference in the success rate among groups A, B and C (χ^2^=7.370; P=0.025). The S-N-K paired comparison showed that the success rate was significantly different between groups A and B (χ^2^=6.452; P=0.011) and between groups B and C (χ^2^=5.240; P=0.022), but not between groups A and C (χ^2^=0.072; P=0.789). The CCA insertion was associated with a higher success rate than the ECA insertion in each group, however, there was no significant difference between the two insertion methods. The success rate of the models subjected to incisions in the middle of the neck corresponded to that of those subjected to lateral neck incisions [A1-(1) vs. A1-(2), χ^2^=0.311, P=0.577; A2-(1) vs. A2-(2), χ^2^=0.311, P=0.577; B1-(1) vs. B1-(2), χ^2^=0.000, P=1.000; B2-(1) vs. B2-(2), χ^2^=1.077, P=0.299; C1-(1) vs. C1-(2), χ^2^=0.000, P=1.000; C2-(1) vs. C2-(2), χ^2^=1.167, P=0.288].

## Discussion

The MCAO model is a typical model widely used in studies on cerebral infarction (13,14). MCAO-induced ischemia-reperfusion injury in the mammalian cerebrum resembles that in humans (15,16), therefore, this animal model has been widely used to study ischemic brain diseases (4,17,18). The disadvantages of the model are its low success rate and the high mortality that results from a number of factors that may affect it, including gender, age, strain (17,19), body temperature (20,21), weight, anesthetics (22,23), blood pressure, blood gas (24), occluding suture size and shape, ligation, insertion (25) and post-operative care. Occluding sutures with varying tip diameters ranging from 0.18–0.30 mm have been used in previous studies (5–7) as even the size of the line tip is taken into consideration during MCAO surgery. The effect of these factors, as well as artery ligation and incision, on the success rate of the MCAO model has not been studied systematically thus far. Previous reports showed that the diameter and length of the MCA were positively correlated with the body weight of the rat, indicating that body weight is critical when creating the MCAO model (26). Animals >350 g in weight have a wider MCA, which makes it difficult to block the blood flow. By contrast, the occluding suture is not easily be inserted into the MCA of rats weighing <240 g. Therefore, rats weighing 250–300 g are suitable for use in an MCAO model, as in the present study.

The infarcted area is a critical factor in assessing therapeutic effects in cerebral infarction cases. An ideal animal model should be stable for repetition; MCAO surgery should generate a stable model of cerebral infarction with consistent ischemia. However, in clinical cases, the size and location of the infarcted region are not consistent, which results in varying effects in animal and clinical experiments (27). Although laser-doppler scanning of the cerebral blood flow was suggested to effectively predict the infarct volume (28), the extent of the infarct was directly evaluated by TTC in the present study. The study demonstrated that the cerebral infarction rates in groups A, B and C were 20.723±1.419, 21.063±1.163 and 21.614±1.067%, respectively; these values were significantly different among the three groups (F=7.024; P=0.001). A paired comparison showed that the cerebral infarction rate in groups A and C or B and C, were significantly different (P<0.05), while the rate in groups A and B was not significantly different (P>0.05). There were no significant differences in the cerebral infarction rate between the ECA and CCA insertion subgroups of the 3 groups nor between the smaller middle and lateral neck incision subgroups of the ECA or CCA (P>0.05). Group C, with the largest diameter (0.28–0.29 mm), had the highest cerebral infarction rate of the three groups. These results indicate that the cerebral infarction volume in models weighing 250–300 g varies with the diameter of the occluding suture or its tip. The RSD of the cerebral infarction rate decreased when the diameter of the line became larger. The RSD of type 1.0, 2.0 and 3.0 lines were 6.85, 5.52 and 4.94%, respectively, depending on whether the diameter of the line coincided with the ICA or the size of the tip coincided with the anterior cerebral artery as the artery may be relatively plastic when the occluding suture is inserted. The occluding suture blocks blood flow only if it is suitable for the artery. In such situations, the infarcted volume steadily increases in size. If the occluding suture is unsuitable, the blood flow is not blocked completely and the infarcted volume also becomes unstable. Other factors, including the material, insertion depth, damage to the artery or brain, inflammation, thrombosis, hemorrhage and individual differences, may affect the cerebral infarction volume. The results of the present study indicated that an occluding suture with a larger diameter was able to give rise to a more stable infarction volume in this MCAO model. However, a stable infarcted volume is not the only criteria for an ideal MCAO-induced cerebral infarction model, which should also simulate the pathology of clinical cases. The model must have a high success rate and the surgery must be convenient. The nervous behavior scores in clinical cases improve with the course of the disease. The present study showed that Zea-Longa scores of nervous behavior in group A (type 1.0 line) did not differ among the 5 time-points (F=0.393; P=0.814), but differed markedly in groups B (type 2.0 line; F=16.315; P=0.000) and C (type 3.0 line; F=16.315; P=0.000). HE staining indicated a typical pathology among the brains of animals in group B (type 1.0 line). These results suggested that the suture diameter was able to directly and/or indirectly affect the clinical pathophysiological features. The lack of a difference in group A (type 2.0) may be the result of an incomplete blockage and a small infarction volume.

Compared with group B (type 1.0), groups A (type 2.0) and C (type 3.0) had lower success rates. The reasons for this may be that: in group A, the diameter of the occluding suture was not large enough to block the blood flow, and the line was too soft and easy to curve into the pterygopalatine artery (PPA); and in group B, the line was too hard and thick and it was difficult to control the size of the tip. The insertion surgery readily leads to vasospasms, which make it difficult to control the insertion force and may lead to SAH. The line tends to pierce into brain tissue, which may result in severe infarction, epilepsy and cerebral hernia. As the nervous behavior scores and infarction rate were higher in group C than those in the other two groups, this demonstrated that the MCAO-induced injury in group C was more severe. The curves of the nervous behavior scores showed that group A was stable while group C changed greatly.

**Figure 4:**
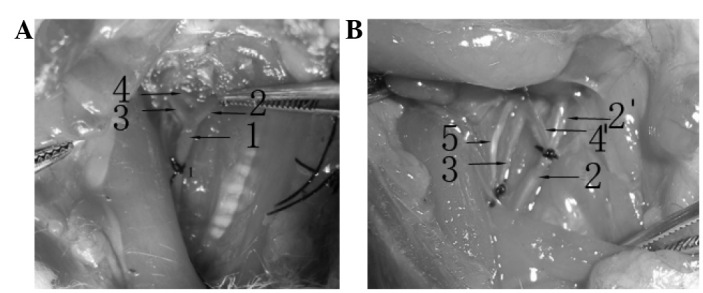
(A) The CCA usually divides into the ICA and ECA at the level of the sternocleidomastoid and sternohyoid muscles with one branch originating from the ECA and linked to the ICA. (B) Variations in the branch point of the ICA and ECA were observed. The branch point was inferior to the normal branch point. Four branches originated from the ECA and two were linked to the ICA. CCA, common carotid artery; ICA, internal carotid artery; ECA, external carotid artery; 1, CCA; 2, ECA; 2′, ECA variation; 3, ICA; 4, branch from the ECA; 4′, variation branch from the ECA; 5, vagus nerve.

ECA insertion is a conventional and widely used method, which swerves the normal artery, therefore, passive distraction leads to excitation of the vagus nerve, which is harmful to the cerebral infarction. The segment of the line remaining outside of the ECA continuously changes the normal artery and results in a lasting distraction. However, CCA insertion appears to be much more simple than ECA as it does not alter the normal direction of the artery. Furthermore, the sinuosity between the CCA and ICA is limited and the CCA is larger than the line diameter. The rapid nature of the surgery and the brief duration of exposure also contribute to the high success rate. Variations in artery insertion have rarely been studied, particularly in the CCA (Fig. 4). An occluding suture is made by knotting the line. The intravascular nylon suture is 18±2 mm long so that the segment behind the knot may be either short for permanent cerebral infarction models or long for ischemia-reperfusion cerebral infarction models. Subsequent to ligation, the segment outside the artery forms an arch, which prevents insertion into the PPA. Consistent with a previous study (29), the PPA was exposed but not intercepted in the present study.

In summary, the results of the present study suggest that the suture diameter and insertion route are able to affect the infarct volume and success rate in the preparation of suture MCAO rat models. However, a limitation of this study was that the cerebral infarction and edema rate were only detected at 72 h post-surgery as the model animals in group B were used for further pharmacological experiments. If the detailed time-dependent changes were determined accurately at 4, 8, 24, 48 and 72 h subsequent to the MCAO operation, the results should reveal the subtle differences between the groups and provide an improved model for the clinic. The data suggest that the suture diameter and insertion route may be associated with the change in cerebral infarction and the success rate, however, the exact mechanism is not clear. The disturbance of blood from the ICA to the PCA (posterior cerebral artery) may affect the size of the infarction (30). Thus, the results would be more rigorous if cerebral blood flow was monitored. Considering that a large sample size is likely to diminish the interference of factors to a certain degree, the results of the present study may be reliable as a total of 84 rats were used.
